# Association of healthy lifestyle with coronary artery disease risk varies by weight status: a prospective cohort study

**DOI:** 10.3389/fnut.2026.1854100

**Published:** 2026-07-06

**Authors:** Fengxu Zhang, Zhengfang Wang, Han Zhang

**Affiliations:** Health Management Center, Beijing Aerospace General Hospital, Beijing, China

**Keywords:** coronary artery disease, healthy lifestyle, metabolic health, obesity, weight status

## Abstract

**Background:**

A healthy lifestyle reduces the risk of coronary artery disease (CAD). However, whether this observed association varies by weight status remains unclear. This study aimed to evaluate the relationship between a healthy lifestyle and the risk of CAD across different weight statuses.

**Methods:**

This prospective cohort study included 8,787 Chinese adults recruited from a health examination center. We developed a healthy lifestyle score (0–4 points) based on smoking, drinking, physical activity, and diet. Participants were classified as normal weight (18.5 ≤ BMI < 24.0) or obesity (BMI ≥ 28 kg/m^2^). The primary outcome was the incidence of CAD. Cox proportional hazards regression models were used to estimate hazard ratios (HR) and 95% confidence intervals (CI), stratified by weight status, and interaction effects were assessed.

**Results:**

Over a median follow-up period of 7 years, 476 CAD events occurred. Each 1-point increase in lifestyle score was associated with a 24% reduction in CAD risk (HR = 0.76, 95% CI: 0.68–0.86). Stratified analysis revealed a significant inverse association in normal-weight individuals (HR = 0.75, 95% CI: 0.65–0.86), while the association in the obesity group did not achieve statistical significance (HR = 0.85, 95% CI: 0.67–1.07). The interaction between lifestyle score and weight status was not statistically significant (*p* for interaction = 0.464). Joint effect analysis indicated that obese individuals with an unhealthy lifestyle exhibited the highest CAD risk (HR = 1.58, 95% CI: 1.13–2.21).

**Conclusion:**

A healthy lifestyle is significantly associated with a reduced risk of CAD among normal-weight individuals. In contrast, this association is not statistically significant in obese individuals, suggesting potential heterogeneity in the relationship between healthy lifestyle scores and CAD risk based on weight status. Given the observational design and limited power in some subgroup analyses, the results should be interpreted as hypothesis-generating and require confirmation in larger prospective studies.

## 1 Introduction

Coronary artery disease (CAD) remains a leading cause of morbidity and mortality worldwide. In 2019, approximately 197 million individuals were affected by CAD globally, resulting in 9 million deaths annually, which represents over 45% of all cardiovascular fatalities (1, 2). In China, the prevalence of CAD has steadily increased in recent decades, driven by rapid urbanization, lifestyle changes, and an aging population (3–6). According to the China Cardiovascular Health and Disease Report, more than 11 million patients are diagnosed with CAD, accounting for a significant proportion of annual disability-adjusted life years lost (7). Given this escalating public health challenge, the identification of effective preventive strategies is of paramount importance.

A healthy lifestyle—characterized by not smoking, moderate or no alcohol intake, regular physical activity, and a healthy diet—has been shown to significantly reduce the risk of CAD (8, 9). The INTERHEART study revealed that lifestyle factors contribute to nearly 90% of the population-attributable risk for myocardial infarction, underscoring the critical role of behavioral modification (10). The American Heart Association has recently introduced the Life’s Essential 8 framework, which quantifies cardiovascular health based on four health behaviors and four health factors, thereby reinforcing the notion that a healthy lifestyle offers substantial cardiovascular benefits (11). Despite the well-established overall association between lifestyle and health outcomes, it remains uncertain whether these benefits are uniformly applicable across different weight statuses.

Obesity is a recognized risk factor for CAD, yet its relationship with cardiovascular outcomes is complex. Two distinct obesity phenotypes have been identified: metabolically healthy obesity (MHO), characterized by a favorable metabolic profile despite high adiposity, and metabolically unhealthy obesity (MUO), which is associated with multiple metabolic abnormalities such as hypertension, dyslipidemia, and hyperglycemia (12, 13). Accumulating evidence indicates that the cardiovascular risk associated with obesity is heterogeneous and that metabolic health status may influence the efficacy of lifestyle interventions (14, 15). Previous studies have demonstrated that the beneficial effects of a healthy lifestyle on cardiovascular outcomes may be diminished in individuals with obesity, particularly among those with established metabolic disturbances (16–18). Furthermore, prior research has predominantly focused on Western cohorts with a high prevalence of obesity, which may limit the applicability of their findings to Asian populations. Our study contributes to the existing literature by employing China-specific obesity criteria, assessing a graded lifestyle score, and formally testing for multiplicative interactions.

Therefore, the present study was designed to assess the association between the composite healthy lifestyle score and incident CAD in a large prospective cohort of Chinese adults, as well as to determine whether this association varies by weight status. Additionally, we investigated the combined effect of weight status and lifestyle on CAD risk and conducted exploratory analyses across metabolic-obesity phenotypes. We hypothesized that a healthy lifestyle would reduce the risk of CAD and that this association would be more pronounced in individuals of normal weight compared to those who are obese, thereby highlighting the potential importance of early lifestyle interventions prior to the onset of obesity.

## 2 Materials and methods

### 2.1 Participants

**Figure 1:**
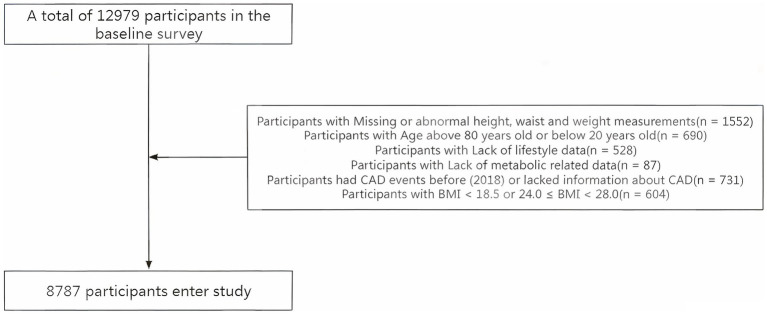
Study flow diagram illustrating the participant selection process.

A prospective cohort study was conducted, recruiting participants from individuals who underwent health examinations at a hospital-affiliated health examination center in 2018. Participants free of CAD and severe hepatic or renal dysfunction at baseline were included. Baseline data were collected through standardized questionnaires, physical examinations, and laboratory tests. All participants were prospectively followed until the occurrence of a CAD event or the study endpoint. A total of 12,979 adults aged 18 years or older underwent health examinations between January 1 and December 31, 2018. Participants were excluded for the following reasons: (1) Missing or abnormal height, waist and weight measurements (*n* = 1,552). (2) Age above 80 years old or below 20 years old (*n* = 690). (3) Lack of lifestyle data (*n* = 528). (4)Lack of metabolic related data (*n* = 87). (5) People who had CAD events before (2018) or lacked information about CAD (*n* = 731) (6) BMI < 18.5 or 24.0 ≤ BMI < 28.0 (*n* = 604). We excluded the overweight group (24.0 ≤ BMI < 28.0 kg/m^2^) from the primary analysis to maximize the contrast between normal weight and obesity, thereby providing a clearer assessment of effect modification by extreme weight status, which may limit the generalizability of our primary findings to overweight individuals. Therefore, we conducted a sensitivity analysis by redefining the normal-weight group to include overweight participants, and the results remained consistent (Supplementary Table S1), supporting the robustness of our conclusions across different weight classifications. The detailed inclusion and exclusion process is shown in Figure 1. The final analysis included 8,787 participants. The study protocol was reviewed and approved by the Institutional Review Board of Beijing Aerospace General Hospital [Approval No: (2025)clinical(25)], and all subjects provided written informed consent.

### 2.2 Data collection and variable definitions

Baseline data were collected using standardized health examination procedures and structured questionnaires. All measurements were conducted by trained medical personnel in accordance with established protocols.

Anthropometric measurements were conducted using calibrated equipment, with participants attired in light clothing and without shoes. Body Mass Index (BMI) was calculated by dividing weight in kilograms by the square of height in meters (kg/m^2^). Waist circumference was measured at the midpoint between the lowest rib and the iliac crest. Blood pressure was recorded twice after a minimum of 10 min of rest, utilizing a standard mercury sphygmomanometer. The average of these two readings was employed for analysis. Hypertension was classified as a systolic blood pressure of ≥140 mmHg, a diastolic blood pressure of ≥90 mmHg, a self-reported history of hypertension, or the current use of antihypertensive medications (19).

#### 2.2.1 Laboratory measurements

Venous blood samples were collected following an overnight fast of at least 10 h. Fasting plasma glucose (FPG), total cholesterol (TC), high-density lipoprotein cholesterol (HDL-C), low-density lipoprotein cholesterol (LDL-C), and homocysteine (HCY) levels were measured using standardized enzymatic methods on an automated biochemical analyzer (Hitachi 7,600, Tokyo, Japan). Diabetes mellitus was defined as an FPG level of ≥7.0 mmol/L, a self-reported history of diabetes, or current use of glucose-lowering medications (20).

#### 2.2.2 Lifestyle factors

Information regarding smoking status, alcohol consumption, physical activity, and dietary habits was collected using a validated self-administered questionnaire. Smoking status was categorized into three groups: never smokers, former smokers, and current smokers. For the purpose of the lifestyle score, both never and former smokers were considered to have a healthy status. Alcohol consumption was similarly classified into three categories: never drinkers, former drinkers, and current drinkers. Non-drinkers (both never and former) were deemed to have a healthy status. Physical activity was evaluated based on the frequency and duration of moderate- or vigorous-intensity exercise; regular physical activity was defined as engaging in moderate-intensity exercise for a minimum of 150 min per week or vigorous-intensity exercise for at least 75 min per week (21).

#### 2.2.3 Healthy lifestyle score

A healthy lifestyle score was constructed by summing the number of healthy behaviors, with one point assigned for each of the following: (1) non-smoking (never or former smoker); (2) non-drinking (never or former drinker); (3) regular physical activity (meeting the recommended duration); and (4) Dietary habits, which were assessed using a validated food frequency questionnaire that covered five key components aligned with the Chinese Dietary Guidelines (35): ① daily vegetable intake (≥1 serving/day); ② daily fruit intake (≥1 serving/day); ③ whole grains consumption (≥3 times/week); ④ red meat intake (≤3 times/week); and ⑤ fried foods intake (≤1 time/week). Each component was scored as 1 if the criterion was met and 0 otherwise. A healthy diet was defined as a total score of ≥3 (36, 37). The total score ranged from 0 to 4, with higher scores indicating a healthier lifestyle. For categorical analyses, participants were classified into unhealthy (0–2 points) and healthy (3–4 points) lifestyle groups based on the distribution and prior literature.

#### 2.2.4 Metabolic-obesity phenotypes

Metabolic abnormalities were defined in accordance with the 2020 guidelines established by the Chinese Diabetes Society for the diagnosis of metabolic syndrome (20). This definition is based on the following five criteria: Central obesity, indicated by a waist circumference of ≥90 cm in men or ≥85 cm in women; Hyperglycemia, characterized by a FPG level of ≥6.1 mmol/L, self-reported diabetes, or the use of glucose-lowering medication; Hypertension, defined as a blood pressure of ≥130/85 mmHg, self-reported hypertension, or the use of antihypertensive medication; Hypertriglyceridemia, indicated by triglyceride levels of ≥1.7 mmol/L; and Low HDL-C, defined as HDL-C levels of <1.04 mmol/L individuals were defined as those having one or fewer of the specified abnormalities, while those with two or more abnormalities were classified as metabolically unhealthy, in accordance with previous studies.

Obesity was defined as BMI ≥ 28.0 kg/m^2^ according to the Chinese criteria (20), while normal weight was defined as 18.5 ≤ BMI < 24.0 kg/m^2^.

The metabolic-obesity phenotypes were defined as follows: Metabolically healthy normal weight (MHNW), characterized by individuals who are metabolically healthy and maintain a normal weight; Metabolically unhealthy normal weight (MUNW), which includes those who are metabolically unhealthy yet have a normal weight; Metabolically healthy obesity (MHO), referring to individuals who are classified as metabolically healthy while being obese; Metabolically unhealthy obesity (MUO), which encompasses those who are both metabolically unhealthy and obese.

### 2.3 Outcome ascertainment

The primary outcome was incident CAD, defined as a composite of non-fatal myocardial infarction, coronary revascularization (including percutaneous coronary intervention or coronary artery bypass grafting), or death attributable to CAD. Cases were confirmed through a review of medical records and International Classification of Diseases (ICD-10) codes (I20-I25). Participants were monitored from the date of the baseline examination until the occurrence of CAD, death, loss to follow-up, or December 31, 2025, whichever came first.

### 2.4 Covariates

Potential confounding factors were collected, including age, gender, and education level. Laboratory covariates included HCY and LDL-C. Variables that served as potential mediators, such as blood pressure, FBG, triglycerides, HDL-C, as well as components of the exposure or stratification variables, including BMI and waist circumference, were not adjusted in the primary models to avoid over-adjustment bias.

### 2.5 Quality control

Prior to the investigation, all field investigators shall undergo uniform training, standardize the physical examination methods, and conduct the field investigation using unified instruments and standardized procedures. During the investigation, inspectors will be appointed to perform random checks and reviews of the investigation results. Additionally, the findings will be summarized on the same day to promptly address any issues that arise during the investigation.

### 2.6 Statistical methods

Statistical analyses were conducted using SPSS 25.0 and R 4.1.3. A two-sided *p* < 0.05 was considered statistically significant. Continuous data are presented as mean ± standard deviation or median (inter-quartile range), depending on the normality assessed by the Shapiro–Wilk test. Categorical data are presented as frequencies (percentages). Comparisons between groups were performed using Student’s t-test for normally distributed continuous variables, Wilcoxon rank-sum test for non-normally distributed continuous variables, and chi-square test for categorical variables. Cox proportional hazards regression models were utilized to estimate hazard ratios (HR) and 95% confidence intervals (CI) for the association between the healthy lifestyle score and the incidence of CAD. Confounding factors were adjusted in three sequential models: Model 1 adjusted for age and sex; Model 2 additionally adjusted for education level; and Model 3 further adjusted for HCY and LDL-C. Stratified analyses were conducted based on weight status (normal weight vs. obesity) and by metabolic-obesity phenotypes (MHNW, MUNW, MHO, MUO). Multiplicative interaction was tested by including cross product terms in the Cox model, and the *p* for interaction was reported. Joint effect analysis was conducted by combining weight status and lifestyle category into a four-level variable, with normal weight plus healthy lifestyle as the reference. To evaluate the robustness of our primary findings regarding the exclusion of overweight participants, we conducted a sensitivity analysis by redefining the normal-weight group to include overweight individuals (24.0 ≤ BMI < 28.0 kg/m^2^) and re-examined the association using the same Cox models (Models 1–3).

## 3 Results

### 3.1 Basic characteristics of the participants

A total of 8,787 participants were included in this study, consisting of 7,342 normal weight and 1,445 obese individuals. Compared to the normal-weight group, obese participants exhibited a higher proportion of males (75.8% vs. 62.8%, *p* < 0.001) and lower lifestyle scores (3.0 ± 0.9 vs. 3.3 ± 0.8, *p* < 0.001), as well as a lower prevalence of healthy lifestyle habits (69.7% vs. 81.1%, *p* < 0.001). Furthermore, obese individuals demonstrated higher levels of BMI, waist circumference, blood pressure, fasting glucose, and LDL-C, along with lower levels of HDL-C (*p* < 0.001). The prevalence of hypertension (56.1% vs. 31.2%) and diabetes (19.7% vs. 11.4%) was significantly greater in the obesity group (*p* < 0.001). Among normal-weight participants, 42.4% were classified as MHNW, while 57.6% were MUNW. In contrast, the obesity group comprised 89.3% MHO individuals and 10.7% MUO individuals. During a median follow-up period of 7 years, 476 CAD events occurred, with a significantly higher incidence rate observed in the obesity group (12.19 vs. 7.14 per 1,000 person-years, *p* < 0.001) (Table 1).

**Table 1:** Basic characteristics of the study population by weight status (*N* = 8,787).

Characteristic	Total (*N* = 8,787)	Normal weight (*n* = 7,342)	Obesity (*n* = 1,445)	*p* value
Age, years	51.0 ± 13.5	50.9 ± 13.6	51.4 ± 12.8	0.121
Male, *n* (%)	5,704 (64.9)	4,609 (62.8)	1,095 (75.8)	<0.001
Education, *n* (%)				
Middle or below	861 (9.8)	657 (9.0)	204 (14.1)	<0.001
Secondary	1,606 (18.3)	1,302 (17.7)	304 (21.1)	
College	3,731 (42.5)	3,115 (42.5)	616 (42.7)	
Graduate or above	2,584 (29.4)	2,264 (30.9)	320 (22.2)	
Lifestyle score	3.2 ± 0.8	3.3 ± 0.8	3.0 ± 0.9	<0.001
Lifestyle category, *n* (%)				
Unhealthy (0–2)	1824 (20.8)	1,386 (18.9)	438 (30.3)	<0.001
Healthy (3–4)	6,963 (79.2)	5,956 (81.1)	1,007 (69.7)	
BMI, kg/m^2^	24.8 ± 3.4	21.8 ± 2.5	30.3 ± 2.3	<0.001
Waist circumference, cm	85.3 ± 9.6	82.8 ± 7.9	97.8 ± 7.4	<0.001
SBP, mmHg	128 ± 20	126 ± 20	136 ± 19	<0.001
DBP, mmHg	76 ± 12	75 ± 12	82 ± 12	<0.001
Hypertension, *n* (%)	3,245 (36.9)	2,288 (31.2)	810 (56.1)	<0.001
FBG, mmol/L	6.0 ± 1.4	5.9 ± 1.4	6.3 ± 1.6	<0.001
Diabetes, *n* (%)	1,234 (14.0)	837 (11.4)	284 (19.7)	<0.001
TC, mmol/L	5.2 ± 1.0	5.2 ± 1.0	5.2 ± 1.0	0.990
HDL-C, mmol/L	1.4 ± 0.3	1.4 ± 0.3	1.2 ± 0.2	<0.001
LDL-C, mmol/L	3.1 ± 0.8	3.1 ± 0.8	3.2 ± 0.8	<0.001
HCY, μmol/L	13.6 (10.9, 17.3)	13.5 (10.8, 17.1)	14.1 (11.4, 18.5)	<0.001
Metabolic healthy, *n* (%)	4,406 (50.1)	3,115 (42.4)	1,291 (89.3)	<0.001
CAD events				
Person-years of follow-up	59,848	50,171	9,677	<0.001
Number of CAD events	476	358	118	
Incidence rate per 1,000 person-years	7.95	7.14	12.19	

BMI, body mass index; SBP, systolic blood pressure; DBP, diastolic blood pressure; FBG, fasting blood glucose; TC, cholesterol; HDL-C, high density lipoprotein-cholesterol; LDL-C, low density lipoprotein-cholesterol; HCY, homocysteine; *p* ≤ 0.05 was considered statistically significant.

### 3.2 Association between healthy lifestyle score and risk of CAD

The healthy lifestyle score was significantly associated with a reduced risk of CAD. In the fully adjusted model (Model 3), each 1-point increase in the lifestyle score corresponded to a 24% risk reduction in CAD risk (HR = 0.76, 95% CI: 0.68–0.86, *p* < 0.001). Compared to the unhealthy lifestyle group (0–2 points), participants adhering to a healthy lifestyle (3–4 points) exhibited a 28% lower risk (HR = 0.72, 95% CI: 0.59–0.88, *p* = 0.002), demonstrating a significant linear trend (*p* for trend = 0.002). These associations remained robust after adjusting for age, sex, education, HCY, and LDL-C (Table 2).

**Table 2:** Association between healthy lifestyle score and risk of CAD.

Lifestyle score	Model 1	Model 2	Model 3
Continuous (per 1-point increase)			
HR (95% CI)	0.73 (0.65–0.82)	0.76 (0.67–0.85)	0.76 (0.68–0.86)
*p*	<0.001	<0.001	<0.001
Categorical			
Unhealthy (0–2)	1.00 (Ref)	1.00 (Ref)	1.00 (Ref)
Healthy (3–4)	0.72 (0.58–0.87)	0.72 (0.59–0.88)	0.72 (0.59–0.88)
*p* for trend	0.001	0.001	0.002

Model 1, adjusted for age and sex; Model 2, additionally adjusted for education level; Model 3, further adjusted for HCY and LDL-C.

### 3.3 Stratified association between lifestyle score and CAD by weight status and metabolic-obesity phenotypes

**Figure 2:**
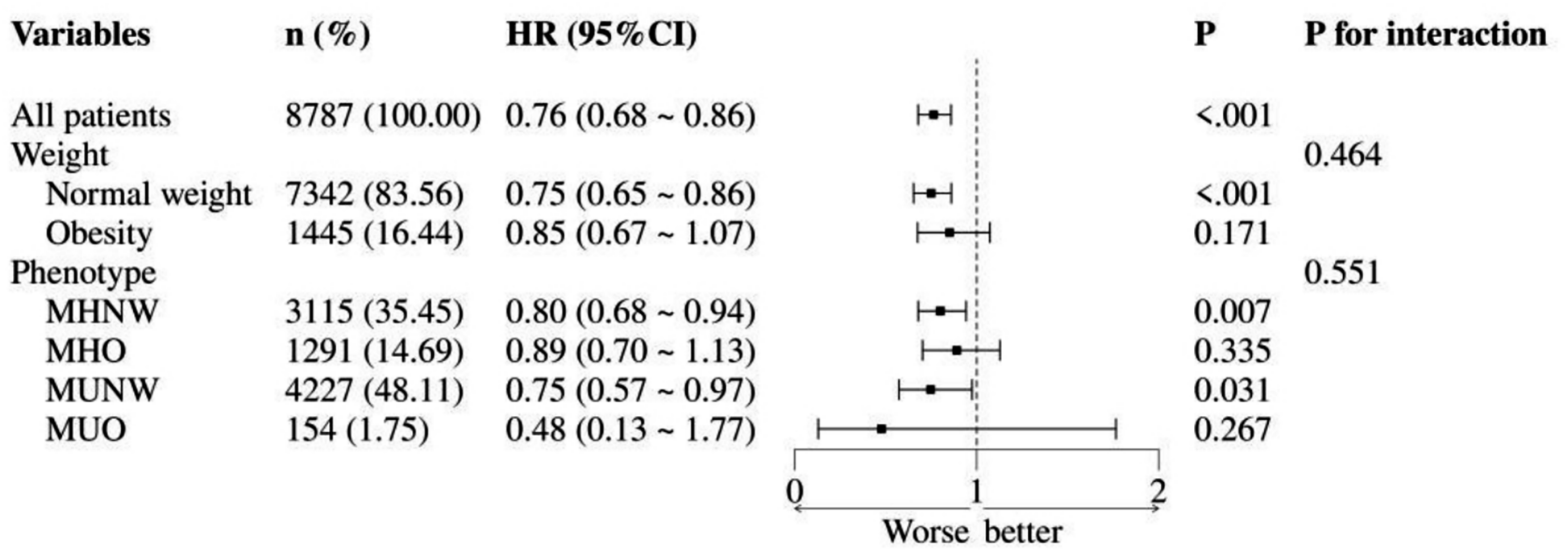
Forest plot summarizing stratified analyses by weight status and metabolic-obesity phenotypes with corresponding HR and interaction *p* values. *The MUO subgroup had limited statistical power (*n* = 154, 7 events); results for this subgroup are exploratory and should be interpreted with caution. The primary stratified analysis combining MHO and MUO into a single obesity group.

In stratified analyses, a higher lifestyle score was significantly associated with a lower risk of CAD among normal-weight individuals (HR = 0.75, 95% CI: 0.65–0.86, *p* < 0.001). However, no significant association was observed in the obesity group (HR = 0.85, 95% CI: 0.67–1.07, *p* = 0.171). The interaction between lifestyle score and weight status was not statistically significant (*p* for interaction = 0.464). In the analysis of metabolic-obesity phenotypes, a higher lifestyle score was significantly correlated with a reduced risk of CAD among MHNW (HR = 0.80, 95% CI: 0.68–0.94, *p* = 0.007) and MUNW (HR = 0.75, 95% CI: 0.57–0.97, *p* = 0.031) individuals. In contrast, no significant association was found in MHO (HR = 0.89, 95% CI: 0.70–1.13, *p* = 0.335) or MUO (HR = 0.48, 95% CI: 0.13–1.76, *p* = 0.267) (Figure 2).

### 3.4 Joint association of weight status and lifestyle with CAD risk

As shown in Table 3, participants with a normal weight but an unhealthy lifestyle exhibited a significantly higher risk of CAD compared to those with a healthy lifestyle in the reference group (HR = 1.34, 95% CI: 1.07–1.69, *p* = 0.012). Among individuals classified as obese, those maintaining an unhealthy lifestyle demonstrated the highest risk (HR = 1.58, 95% CI: 1.13–2.21, *p* = 0.007). In contrast, the risk for obese individuals adhering to a healthy lifestyle was elevated, although not statistically significant (HR = 1.15, 95% CI: 0.87–1.53, *p* = 0.316).

**Table 3:** Joint association of weight status and lifestyle with CAD risk.

Group	Participants	Events	HR (95% CI)	*p*
Normal weight + Healthy lifestyle	5,956	264	1.00 (Ref)	
Normal weight + Unhealthy lifestyle	1,386	94	1.34 (1.07–1.69)	0.012
Obesity + Healthy lifestyle	1,007	81	1.15 (0.87–1.53)	0.316
Obesity + Unhealthy lifestyle	438	37	1.58 (1.13–2.21)	0.007

HR were adjusted for age, sex, education, HCY, and LDL-C.

### 3.5 Sensitivity analysis including overweight participants

To evaluate the potential impact of excluding overweight individuals on our conclusions, we repeated the analysis by including overweight participants (*n* = 604) in the normal-weight group. As shown in Supplementary Table S1, the inverse association between the healthy lifestyle score and CAD risk remained statistically significant, although the effect sizes were slightly attenuated compared to the primary analysis. In the fully adjusted model (Model 3), each 1-point increase in the lifestyle score was associated with a 12% reduction in CAD risk (HR = 0.88, 95% CI: 0.77–0.99, *p* = 0.047). When analyzed categorically, participants with a healthy lifestyle (score 3–4) exhibited a 15% lower risk compared to those with an unhealthy lifestyle (HR = 0.85, 95% CI: 0.72–0.98; *p* for trend = 0.048). These results support the robustness of our primary findings, indicating that the observed association between a healthy lifestyle and CAD risk is consistently observed regardless of the inclusion of overweight individuals in the normal-weight reference group.

## 4 Discussion

Based on a cohort of 8,787 Chinese adults undergoing health examinations, this study systematically evaluated the association between a healthy lifestyle score and the risk of CAD. Furthermore, it explored whether this association varied by weight status and metabolic-obesity phenotypes. A higher healthy lifestyle score was significantly associated with a lower risk of CAD, with each 1-point increase corresponding to a 24% risk reduction. Individuals with a healthy lifestyle (score 3–4) had a 28% lower risk compared to those with an unhealthy lifestyle (score 0–2). This observed association was significant among normal-weight individuals but not among those with obesity, with a non-significant interaction between weight status and lifestyle (*p* for interaction = 0.464). In the analysis of the metabolic-obesity phenotypes, a significant association was observed only in MHNW and MUNW individuals, whereas no significant associations were found in MHO or MUO individuals. It is important to note that the MUO subgroup contained only 154 participants and 7 CAD events; therefore, the null finding in this subgroup is based on very limited statistical power and should be considered exploratory only. Consequently, the primary analysis of obesity-related outcomes therefore relied on the combined obesity group (MHO+MUO) as presented in Figure 2. Joint effect analysis revealed that normal-weight individuals with a healthy lifestyle had the lowest risk, whereas obese individuals with an unhealthy lifestyle exhibited the highest risk (HR = 1.58). These findings suggest that the association between a healthy lifestyle and CAD risk is more pronounced among normal-weight individuals, while the association appeared weaker and did not reach statistical significance among individuals with obesity.

The findings of this study align with those from several large-scale cohort studies. The Nurses’ Health Study indicated that adherence to a healthy lifestyle—characterized by non-smoking, regular physical activity, and moderate alcohol consumption—was associated with an approximately 80% reduction in the risk of CAD (22). More recently, the American Heart Association’s Life’s Essential 8 (LE8) score has been shown to have an inverse association with cardiovascular disease and all-cause mortality across multiple cohorts (23–26). Additionally, the healthy lifestyle score derived from four core lifestyle factors in the present study demonstrated a significant observed association with reduced CAD risk, thereby further supporting the pivotal role of healthy lifestyles in the primary prevention of cardiovascular disease.

Regarding the modifying effect of weight status on the observed association between lifestyle and cardiovascular risk, existing studies have reported inconsistent findings. Some research indicates that adherence to a healthy lifestyle significantly reduces cardiovascular risk even in individuals with overweight or obesity (27–29). Conversely, other studies suggest that obesity may diminish the benefits of a healthy lifestyle, particularly over long-term follow-up. For instance, a study utilizing data from the UK Biobank discovered that the strength of the association between a healthy lifestyle and cardiovascular disease risk was weaker in obese individuals compared to those of normal weight (30). The findings of the present study align more closely with the latter perspective, revealing a significant association of lifestyle in normal-weight individuals, while showing a non-significant effect in those with obesity. Our findings carry several practical implications, which should be considered as suggestive rather than definitive. First, normal-weight individuals, particularly those classified as MUNW, represent a critical window of opportunity for lifestyle intervention. This group, which accounted for nearly half of our normal-weight participants, demonstrated that a healthy lifestyle is associated with a 25% reduction in CAD risk. Given that metabolic abnormalities often precede the onset of obesity, early intervention through smoking cessation, moderation of alcohol consumption, regular physical activity, and adherence to a healthy diet may prevent or delay the progression to obesity and ultimately reduce long-term cardiovascular risk. Second, in individuals with obesity, the observed association between lifestyle score and CAD risk did not reach statistical significance (HR = 0.85; 95% CI: 0.67–1.07). However, this null finding should not be interpreted as evidence that lifestyle modification is ineffective for this population. The lack of statistical significance may be attributed to limited statistical power, residual confounding, measurement error, medication use, or unmeasured lifestyle factors, rather than indicating a true absence of benefit. Therefore, lifestyle modification should continue to be promoted among all individuals, as our findings do not undermine the importance of maintaining healthy lifestyles for obese individuals. Future research with larger sample sizes and more comprehensive measurements is necessary to elucidate the association in this demographic.

In the present study, a 1-point increase in lifestyle score was associated with a 15% reduction in CAD risk within the obesity group (HR = 0.85, 95% CI: 0.67–1.07, *p* = 0.171). However, no significant associations were observed in either the MHO or MUO subgroups in the four-phenotype analysis. Several speculative mechanisms could contribute to the null findings, though none were directly tested in this study. First, obesity itself is associated with multiple pathophysiological alterations, including adipose tissue inflammation, insulin resistance, and endothelial dysfunction. Although speculative in the context of our study, it is possible that such alterations could contribute to long-term vascular changes (31). Beyond these, obesity-induced vascular remodeling - characterized by arterial stiffening, intima-media thickening, and reduced vascular compliance - further compromises cardiovascular health and may not be fully reversible through lifestyle modification alone; however, this remains a hypothesis (32). Moreover, the concept of so-called metabolic memory suggests that prolonged exposure to a metabolically unfavorable environment could induce persistent genetic changes that maintain elevated CAD risk even after risk factors are controlled. In the context of obesity, metabolic memory has been proposed as one possible explanation for persistent cardiovascular risk, but this mechanism was not measured in the present study and should be interpreted cautiously. These potential mechanisms may be particularly relevant in Chinese populations, who exhibit a higher susceptibility to central obesity and insulin resistance at lower BMI levels compared to Western counterparts. This “metabolic susceptibility” means that even modest weight gain could trigger significant metabolic disturbances, potentially accelerating vascular damage and creating a metabolic memory effect earlier in the disease course. If this hypothesis is correct, then by the time obesity is established, longer exposure to adverse metabolic conditions may partly explain the weaker observed association in individuals with obesity, although this hypothesis was not directly tested in the present study. This interpretation aligns with our finding that the observed association of a healthy lifestyle was not statistically significant in obese individuals, whereas it was clearly evident in normal-weight individuals, especially those with metabolically unhealthy normal weight. Second, confounding by medication use may play a significant role. Obese individuals exhibit a higher prevalence of hypertension, diabetes, and dyslipidemia (56.1 and 19.7% in the present study, respectively), leading to an increased likelihood of using antihypertensive, glucose-lowering, and lipid-lowering medications (32). These medications provide substantial cardiovascular protection, which may obscure or diminish the independent effects of lifestyle factors. Third, while the healthy lifestyle score utilized in this study encompasses four core lifestyle factors, it fails to include other dimensions recognized as important for cardiovascular health, such as sleep quality, psychological stress, and sedentary behavior. Among obese individuals, poor sleep quality and elevated psychological stress are more prevalent, potentially reducing the predictive power of conventional lifestyle metrics (33, 34).

In the normal-weight group, a significant inverse association between lifestyle score and CAD risk was observed (HR = 0.75), and the joint effect analysis showed that normal-weight individuals with a healthy lifestyle had the lowest risk. Notably, even among MUNW individuals who accounted for 57.6% of the normal-weight group in this study, a healthy lifestyle was associated with significant benefit (HR = 0.75). This finding has important public health implications: lifestyle intervention may be particularly effective in preventing CAD when implemented before the development of obesity, even in the presence of metabolic abnormalities.

The findings of this study have several implications for the primary prevention of cardiovascular disease. A stratified prevention strategy may be warranted. For individuals with a normal weight, promoting a healthy lifestyle should be emphasized, as it confers clear cardiovascular benefits even in those with metabolic abnormalities. For individuals with obesity, the association between lifestyle score and CAD risk was not statistically significant in this cohort. This finding should not be interpreted as evidence that lifestyle modification is ineffective; rather, it may reflect limited power, residual confounding, medication use, measurement error, or unmeasured lifestyle factors. Lifestyle modification should continue to be encouraged, and further studies are needed to determine whether additional risk-management strategies provide incremental benefit. Consequently, identifying normal-weight individuals with metabolic abnormalities during health examinations and prioritizing them for lifestyle intervention could constitute an effective strategy. The simple four-item lifestyle score utilized in this study demonstrated good risk discrimination among normal-weight individuals and could serve as a practical tool for the rapid assessment of cardiovascular health in primary care settings.

Our findings should be interpreted within the context of population differences. Compared to Western cohorts, our study exhibits several distinctive features. First, we employed Chinese obesity criteria (BMI ≥ 28 kg/m^2^), which is lower than the Western threshold (BMI ≥ 30 kg/m^2^), indicating a higher cardiometabolic risk at lower BMI levels in East Asians. Second, the dietary patterns in our cohort are characterized by a higher carbohydrate intake and lower fat consumption compared to typical Western diets, which may influence the association between lifestyle and CAD. Third, the baseline prevalence of obesity in our cohort (16.4%) is significantly lower than that observed in Western populations (typically 30–40%); however, the association between a healthy lifestyle and CAD remained evident even among individuals of normal weight.

The main strengths of this study are as follows: (1) a large cohort of Chinese adults undergoing health examinations; (2) the application of internationally recognized criteria for identifying metabolic abnormalities; (3) a systematic assessment of the effect modification by weight status through stratified analysis and interaction testing; and (4) a joint effect analysis that provides quantitative evidence for clinical decision-making. However, several limitations must be acknowledged. First, the single-center design and reliance on a health examination population may introduce selection bias. As a result, the study population may not fully represent the general Chinese population, given that individuals attending health check-ups tend to be more health-conscious and have better access to healthcare services. This selection bias may limit the generalizability of our findings. Therefore, future validation in large-scale, multi-center, community-based prospective cohorts is necessary to confirm the external validity of our conclusions. Second, lifestyle data were self-reported at baseline, which may be subject to recall bias, and changes in lifestyle over time were not captured. Third, the healthy lifestyle score has several limitations. The binary scoring of each lifestyle component (healthy vs. unhealthy) may oversimplify complex behaviors and overlook important dose–response relationships. For instance, the definition of a healthy diet was based on meeting at least three out of five binary criteria, without quantifying actual intake amounts or distinguishing between food quality. This approach could lead to non-differential misclassification, potentially attenuating the observed associations. Furthermore, the score did not incorporate other relevant lifestyle factors known to influence cardiovascular health, such as sleep quality, psychological stress, and sedentary behavior, which may be particularly significant in the context of obesity. Although we adjusted for several confounders, residual confounding from these unmeasured factors cannot be disregarded. Future studies should adopt more comprehensive and quantitative lifestyle assessments, including the AHA Life’s Essential 8, to capture the full spectrum of health behaviors. Fourth, lifestyle behaviors were assessed only at baseline and were assumed to remain constant throughout the 7-year follow-up period. It is plausible that some participants modified their lifestyles during follow-up, either spontaneously or in response to health recommendations or disease diagnoses. This could have introduced information bias, potentially leading to an underestimation or overestimation of the true association between lifestyle and CAD risk. Future studies should incorporate repeated lifestyle assessments to capture dynamic changes and their effects on CAD risk. Fifth, despite thorough adjustments for a wide range of potential confounders, residual confounding may still persist. Specifically, we were unable to adjust for family history of coronary heart disease, as this information was not collected in the health examination database. Additionally, detailed data on the use of lipid-lowering, glucose-lowering, and antihypertensive medications were not available. Although such medications are likely to be more frequently prescribed to individuals with metabolic abnormalities and obesity, which may influence CAD risk, their absence from the models could result in residual confounding. Future studies should aim to collect comprehensive medication histories and family history data to better control for these factors. Additionally, we excluded overweight participants from the primary analysis to enhance the contrast of our findings. However, sensitivity analyses that included overweight participants yielded consistent estimates (HR per 1-point increase = 0.88, 95% CI: 0.77–0.99), indicating that our main conclusions are not substantially altered. Nonetheless, future studies should specifically investigate the lifestyle-CAD association in overweight populations.

## 5 Conclusion

In conclusion, among Chinese adults undergoing health examinations, a healthy lifestyle was significantly associated with a lower risk of CAD. This association was particularly evident in individuals of normal weight, while it did not achieve statistical significance in obese individuals. These findings suggest potential heterogeneity in the relationship between healthy lifestyle scores and CAD risk based on weight status. However, due to the observational nature of the study, the presence of residual confounding, the exclusion of overweight participants from the primary analysis, and limited statistical power in certain subgroups, the results should be regarded as hypothesis-generating rather than definitive. Future large-scale, community-based prospective studies that include repeated lifestyle measurements and comprehensive adjustments for confounders are necessary to validate these preliminary findings.

## Data Availability

The data presented in this study are not publicly available due to privacy and ethical restrictions. Requests to access the datasets should be directed to HZ, 711zyjk@sina.com.
