# Supplementary material for: Association of healthy lifestyle with coronary artery disease risk varies by weight status: a prospective cohort study
Source: Front Nutr. 2026 Jul 6;13:1854100. doi: 10.3389/fnut.2026.1854100 (PMC13381236; doi:10.3389/fnut.2026.1854100)
Supplement: Supplementary file 1 [file Table_1.DOCX]

Table S1. Sensitivity analysis of the association between healthy lifestyle score and CAD risk after including overweight participants into the normal‑weight group

| **Lifestyle score** | **Model 1** | **Model 2** | **Model 3** |
| --- | --- | --- | --- |
| ****Continuous****  ****(per 1-point increase)**** |  |  |  |
| HR (95% CI) | 0.82 (0.71-0.94) | 0.87 (0.76-0.99) | 0.88 (0.77-0.99) |
| *P* | 0.017 | 0.042 | <0.047 |
| ****Categorical**** |  |  |  |
| Unhealthy (0-2) | 1.00 (Ref) | 1.00 (Ref) | 1.00 (Ref) |
| Healthy (3-4) | 0.70 (0.53-0.94) | 0.81 (0.70-0.92) | 0.85 (0.72-0.98) |
| *P* for trend | 0.017 | 0.045 | 0.048 |

Model 1: adjusted for age and sex;

Model 2: additionally adjusted for education level;

Model 3: further adjusted for HCY and LDL-C.
